# Socio-demographic determinants of neonatal mortality in Algeria according to MICS4 data (2012–2013)

**DOI:** 10.4314/ahs.v21i1.45

**Published:** 2021-03

**Authors:** Adel Sidi-Yakhlef, Meryem Boukhelif, Amaria Aouar Metri

**Affiliations:** 1 AboubakrBelkaid University. Laboratory for the Valorization of Human Action, for the Protection of the Environment and Application in Public Health – Tlemcen – Algeria; 2 AboubakrBelkaid University, Faculty of Humanities and Social Sciences – Tlemcen – Algeria

**Keywords:** Neonatal mortality, Algeria, MICS4 data (2012–2013)

## Abstract

**Background:**

Neonatal mortality remains a public health problem in developing countries, including Algeria. Information on this indicator makes it possible to assess government efforts to improve the living conditions of target populations.

**Objectives:**

This study aims to identify some determinants associated with this mortality from data of multiple indicator cluster survey conducted in Algeria in 2012–2013 (mics 4).

**Methods:**

A retrospective case-control study including 1047 cases and 1041 controls. From a logistic regression model, we appreciated the role of different factors, socio-demographic, economic and geographic (Mother's age, level of education, wealth index, area of residence) in newborn survival.

**Results:**

The main factors associated with neonatal mortality were rural residence (p<0.01; OR= 1.3; CI 1.08–1.54), South geographical area (p<0.05; OR=1.5; CI 1.18–1.84), low education level of mother (p<0.01; OR= 2.10; CI 1.35– 3.29), early age of maternal procreation (p<0.001; OR=4.34; CI 2.19– 14.40), the birth rank “7 and over” (<0.01; OR = 1.57; CI 1.13 – 2.44) and the two lowest wealth indices (p <0.001; OR = 2; 1.45–2.62 and p <0.01; OR = 1.66; CI 1.23–2.26).

**Conclusion:**

In addition to the various reproductive health strategies already adopted by the authorities for health promotion and family planning, action should be taken to evaluate their implementation with sustained assistance for disadvantaged people and in risk areas.

## Introduction

Worldwide, more than 3 million newborns die within the first month of life. During this first month, 25 to 50% of all these deaths occur within first 24 hours of life, and 75% during the first week^1^.

Neonatal mortality is one of the main indicators of health state at birth and of socioeconomic and health progress of the country^2^, ^3^. Information on this indicator makes it possible to assess government efforts to improve the living conditions of target populations, as well as the impact of current health programs. This information plays an undeniable role in a country's national development planning.

According to the WHO (World Health Organization) among the 130 million children born worldwide each year, 4 million die during the first 4 weeks of life and 99% of them die in low-resource countries 4, 5. Considerable disparities in child health remain between and within countries 6. To address these disparities, the international community has already set itself goals in 2000 called the Millennium Development Goals and in 2016 with a new Agenda for Sustainable Development to 2030 consisting of 17 goals aimed at reducing child mortality.

African countries have the highest estimated neonatal mortality rate of 45 deaths per 1000 live births compared to 5 deaths in developed countries 7. In Algeria, the stillbirth rate decreased from 21.4‰ to 15.9‰ during the period (1990–2012) 8. The results of the Multiple Indicator Cluster Survey conducted in 2012–2013 (mics4) in Algeria estimated the neonatal mortality rate to be 16 deaths per 1000 live births. The socio-demographic and economic factors (age of the mother, level of education, wealth index, etc.) associated with this mortality are not clearly identified. Given that the MICS survey reports are limited to descriptive analyses on the one hand, and given the scarcity of case-control studies carried out on the entire Algerian population on the other hand, we have taken an initiative to study some socio-demographic, economic and geographic factors that may be associated with neonatal mortality, in the knowledge that understanding and mastering these determinants allows a better guidance of control strategies at the authority level.

## Materials and methods

This is a retrospective case-control study of all deaths recorded in Multiple Indicator Cluster Survey database conducted in 2012–2013 (mics4) by the Algerian Ministry of Health, Population and Hospital Reform, with technical and financial support from UNICEF and a financial contribution from UNFPA. This database is available on the official UNICEF website.

These descriptive surveys allow many countries to produce statistically reliable estimates for a range of indicators in different areas, including health, in order to monitor changes in the situation of children and women, thereby assisting decision makers in development of policies and intervention programs.

In Algeria, the MICS4 survey covered a sample of 28000 households divided into seven geographical areas known as territorial programming space, making it possible to statistically represent the entire Algerian population at the national level and at the level of these territories.

The data used to study neonatal mortality came from the child mortality module of the “Women” questionnaire. During the collection procedure, all non single women of childbearing age (15–49 years) were asked about the number of live births and the number of deaths. For deceased children, it was requested that the age at death be specified (to the day for deaths less than one month old, to the month for deaths less than two years old, and in years for deaths two years or older)^7^. The case group is represented by all newborns who died between 0 and 28 days. We have listed in the database 1047 cases, i.e. 16 deaths per 1000 live births. To obtain a control group comparable to the case group, we randomly selected from the database a representative sample of the overall population reporting no neonatal deaths and equivalent to the case sample, that is 1041 controls (2% of all controls), using SPSS version 21 statistical software. In order to reduce the number of potential confounding factors and to facilitate subsequent statistical analysis, we selected this sample for individual matching by selecting case-control pairs for the confounding factor “sex of newborns”.

In order to better identify the factors most conducive to neonatal mortality, we selected as independent variables (based on knowledge of proven confounding factors) the socio-demographic characteristics of mothers (mother's age at birth, birth rank, previous reproductive interval, mother's education level), economic characteristics (wealth index Quintiles) and geographic characteristics (territorial programming space, place of residence).

Logistic regression was applied to measure the relationship between each independent variable and the risk of death. The results are expressed as odds ratios (ORs), their 95% confidence intervals.

## Results

The neonatal mortality rate was estimated to be 16 deaths per 1000 live births, according to the final report of the Mics4 survey. Table 1 presents the results of the logistic regression that allowed us to identify the factors predictive of neonatal mortality.

**Table I:** Results of the logistic regression of neonatal mortality according to the factors studied

Factor	Control N - %	Case N - %	Odds ratio	95% CI	P-Value
**Area/stratum**					

**Urban**	661(52.29)	603(47.70)	1		
**Rural**	380(46.11)	444(53.88)	1.30	1.08–1.54	<0.01

**Territorial Programming Spaces (TPS)**					

**TPS 1: North Central**	129(50.58)	126(49.41)	1		
**TPS 2: Northeast**	117(56.52)	90(43.47)	0.76	0.52–1.10	NS
**TPS 3: Northwest**	108(51.42)	102(48.57)	0.96	0.66–1.38	NS
**TPS 4: Central Highlands**	178(47.97)	193(52.02)	1.15	0.84–1.59	NS
**TPS 5: Eastern Highlands**	153(52.21)	140(47.78)	0.88	0.63–1.23	NS
**TPS 6: Western Highland**	179(54.74)	148(45.25)	0.84	0.61–1.17	NS
**TPS 7 : South**	177(41.64)	248(58.5)	1.55	1.18–1.84	<0.05

**Mother's level of education**					

**Illiterate**	341(44.86)	419(55.13)	2.10	1.35–3.29	<0.01
**Elementary**	231(49.67)	234(50.32)	1.68	1.05–2.67	NS
**Middle**	228(51.93)	211(48.06)	1.41	0.91–2.26	NS
**Secondary**	184(55.25)	149(44.74)	1.32	0.82–2.13	NS
**university**	57(62.63)	34(37.36)	1		

**Mother'sage at birth**					

**Less than 20 years**	10(27.77)	26(72.22)	4.35	2.19–14.40	
**35 years and over**	1031(50.24)	1021(49.75)	1		<0.001

**Previous reproductive interval**					

**First Birth**	314(46.93)	355(53.06)	1		
**Less than 2 years**	366(49.93)	367(50.06)	0.89	0.72–1.09	NS
**2 years**	66(57.89)	48(42.10)	0.66	0.44–1.07	NS
**3 years**	46(59.74)	31(40.25)	0.62	0.38–1.01	NS
**4 years and over**	249(50.30)	246(49.69)	0.88	0.69–1.11	NS

**Rank of birth**					

**1**	316(47.80)	345(52.20)	1		
**2–3**	438(52.70)	393(47.30)	0.82	0.67–1.01	NS
**4–6**	241(51.27)	229(48.73)	0.86	0.68–1.09	NS
**7 and over**	46(36.50)	80(63.49)	1.57	1.06–2.32	<0.05

**Wealth index quintiles**					

**The poorest**	230(42.51)	311(57.49)	1.95	1.45–2.69	<0.001
**The Second**	206(47.03)	232(52.92)	1.67	1.23–2.26	<0.01
**The Middle**	232(54.07)	197(45.92)	1.24	0.91–1.69	NS
**The fourth**	210(51.98)	194(48.02)	1.35	0.99–1.83	NS
**The richest**	163(59.05)	113(40.95)	1		

Results show that mothers from rural residence are 1.3 times more likely to lose their newborns compared with those from urban residence (p<0.01; CI 1.08–1.53). Similarly, newborns born to mothers residing in the southern geographic region were 1.5 times more likely to die compared with the North-Central reference region (p<0.05; CI 1.18–1.84), whereas there did not appear to be a significant difference between the other regions. Newborns whose mothers have a low level of education (no education) present about two times more risk of death than those with a university education (OR= 2.10; p<0.01; 1.35 – 3.29). Newborns appear to have a 4.34 risk of dying when born to mothers less than 20 years of age (p<0.001; CI 2.18 – 14.40). The previous birth interval does not appear to be a determinant of neonatal mortality based on our results, as there were no statistically significant differences between the four intervals. However, newborns with a birth rank of “ 7 and above” appear to have a 1.57 times greater risk of death than primiparous women (p=0.01; CI 1.13 – 2.44). As for the wealth index quintiles, newborns from households with the “poorest” and “second poorest” indices seem to be 2 and 1.66 times more likely to die compared to the richest ((p<0.001; 1.45–2.62 and p<0.01; CI 1.23–2.26).

## Discussion

Neonatal mortality is an indicator of health status that is widely used to identify health needs and assess medical management. For the five-year period preceding the MICS4 survey (2008–2012), neonatal mortality was 16 deaths per 1000 live births and post-neonatal mortality was 6 deaths per 1000 live births. This rate is relatively low compared to those of several other developing countries such as Mali 57‰ 9, Mauritania 34‰, Sudan 30‰, Cameroon 26% 10. None the less, it is still higher than that of Canada 3.4% 15, France 2.6 ‰, Luxembourg, Sweden, Norway, and the Czech Republic 2‰ 11. The high risk of death of newborns in rural and southern geographic areas observed in our results is consistent with the results of many studies, The majority believe that this is probably due to the living conditions in this environment, such as the poor drinking water supply, the accessibility of sanitary facilities, the lack of specialized medical coverage, the physical work of women in agriculture, and the lack of the various amenities found in the cities 12, 13, 14.

Several studies agree on the close relationship between the high risk of neonatal death and the low educational level of the parents, which is also revealed by our results 15. Low educational attainment can be a barrier to accessing and understanding public health messages about the importance of antenatal and postnatal care. Also, low educational level is very often associated with low socio-economic level, which is a financial limitation in access to such care. Indeed, educated mothers tend to have a good follow-up of pregnancy, a healthy lifestyle, and can recognize the danger signs which often lead to perinatal mortality 16, 17, 18.

We have also found that neonatal mortality is high among women under 20 years of age compared to other age categories. This category consists mainly of primiparous and adolescent girls who are prone to dystocic childbirth. This may suggest that early childbearing has a negative impact on the survival of the baby. In France, Blondel reported that neonatal mortality was high in women under 20 years of age 19, 15, 20.

The duration of the birth interval does not appear to be a determinant of neonatal mortality in our study. However, several studies suggest that intervals of less than two years reduce a woman's degree of recovery of her physiological abilities, resulting in higher mortality than births that follow their eldest child by 3 or 4 years 21, 22. There is no doubt that poverty is a major factor in health disparities. The relationship between mortality and income has been the subject of many studies, and most agree that poverty and poor health go hand in hand 23,24. Our results regarding the close relationship between low wealth index and neonatal mortality are consistent with those of several authors 15, 23, 25, 26.

It should be noted that the variables studied in this study can explain only part of the determinants of neonatal mortality, which belong to social and natural facts that are sensitive and complex to identify. Other morbidity and socio-anthropological factors can help us better understand and identify this painful social phenomenon. Among the limits of our study, it was noted that since the data were collected from persons alive at the time of the survey (women aged 15–49), it was not possible to obtain information on the survival or death of children whose mothers are deceased. In addition, by limiting data collection to only women aged 15–49 at the time of the survey, the information may not be representative for certain periods, such as women aged 50 and above who are no longer eligible at the time of the survey, which may introduce bias in the overall mortality estimate.

Data collected during the 2012–2013 Survey (mics4) provided estimates of levels, trends and differentials of neonatal mortality. These results should help to measure progress towards the goals set by WHO and will be very useful for the development, monitoring and evaluation of population policies and health and preventive health programs.

## Conclusion

In view of our results, factors that appear to be associated with neonatal mortality in Algeria are: rural area of residence, southern geographical region, low level of maternal education, early maternal childbearing age, birth rank “7 and above” and modest socio-economic level.

This study aimed to identify some determinants associated with neonatal mortality that can sometimes be prevented by better monitoring of pregnancy and proper management of childbirth and the newborn by acting on social disparities in health. The importance to be attached to the protection of mothers and children remains one of the priorities of health and social protection policy in Algeria. This work could help health stakeholders to better understand the determinants of neonatal mortality and to take greater action to improve maternal and child health.
